# Is there a rationale for hyperbaric oxygen therapy in the patients with Post COVID syndrome? A critical review

**DOI:** 10.1007/s00406-024-01911-y

**Published:** 2024-11-15

**Authors:** MT Pawlik, G Rinneberg, A Koch, H Meyringer, TH Loew, A Kjellberg

**Affiliations:** 1 Department of Anesthesiology and Intensive Care Medicine, Caritas-Hospital St. Joseph, University of Regensburg, Regensburg, Germany; 2 Institute of Experimental Medicine, Christian-Albrechts-University of Kiel c/o German Naval Medical Institute, Kronshagen, Germany; 3 Department of Physiology and Pharmacology, Karolinska Institutet, Solna, Sweden; 4 Perioperative Medicine and Intensive Care, Medical Unit Intensive Care and Thoracic surgery, Karolinska University Hospital, Stockholm, Sweden; 5 Department of Psychosomatic Medicine, University Hospital Regensburg, Regensburg, Germany

**Keywords:** Brain fog, Chronic fatigue syndrome, COVID-19, SARS-CoV-2, Hypoxia-inducible-factor-1, Hyperbaric oxygen therapy (HBOT), Long COVID, Post COVID condition

## Abstract

The SARS-CoV-2 pandemic has resulted in 762 million infections worldwide from 2020 to date, of which approximately ten percent are suffering from the effects after infection in 2019 (COVID-19) [1, 40]. In Germany, it is now assumed that at least one million people suffer from post-COVID condition with long-term consequences. These have been previously reported in diseases like Myalgic Encephalomyelitis (ME) and Chronic Fatigue Syndrome (CFS). Symptoms show a changing variability and recent surveys in the COVID context indicate that 10–30 % of outpatients, 50 to 70% of hospitalised patients suffer from sequelae. Recent data suggest that only 13% of all ill people were completely free of symptoms after recovery [3, 9]. Current hypotheses consider chronic inflammation, mitochondrial dysfunction, latent viral persistence, autoimmunity, changes of the human microbiome or multilocular sequelae in various organ system after infection. Hyperbaric oxygen therapy (HBOT) is applied since 1957 for heart surgery, scuba dive accidents, CO intoxication, air embolisms and infections with anaerobic pathogens. Under hyperbaric pressure, oxygen is physically dissolved in the blood in higher concentrations and reaches levels four times higher than under normobaric oxygen application. Moreover, the alternation of hyperoxia and normoxia induces a variety of processes at the cellular level, which improves oxygen supply in areas of locoregional hypoxia. Numerous target gene effects on new vessel formation, anti-inflammatory and anti-oedematous effects have been demonstrated [74]. The provision of intermittently high, local oxygen concentrations increases repair and regeneration processes and normalises the predominance of hyperinflammation. At present time only one prospective, randomized and placebo-controlled study exists with positive effects on global cognitive function, attention and executive function, psychiatric symptoms and pain interference. In conclusion, up to this date HBO is the only scientifically proven treatment in a prospective randomized controlled trial to be effective for cognitive improvement, regeneration of brain network and improvement of cardiac function. HBOT may have not only theoretical but also potential impact on targets of current pathophysiology of Post COVID condition, which warrants further scientific studies in patients.

##  Introduction

The worldwide pandemic of coronavirus disease 2019 (COVID-19) has not only led to an acute overload of the worldwide medical system and in particular of intensive care units due to the sometimes life-threatening symptoms in the course of the infection, but also to post-acute sequelae. The acute phase of COVID-19 is characterised by uncontrolled inflammation, increased hypercoagulability with vascular occlusion in central and peripheral vessels, which leads to a high mortality rate [42]. Generalised inflammation in the body with excessive increases in the cytokines IL-1, IL-6 and TNF-α are referred to as a cytokine storm [177]. Microinfarcts in vessels and neuroinflammation are possible causes of local and specific hypoxia in the central nervous system [135]. COVID-19, caused by the severe acute respiratory syndrome coronavirus type 2 (SARS-CoV-2), leads to a symptom complex with more than 200 differentiable symptoms [120]. These are associated with a wide variety of medical complications, which can often persist for weeks to months after the initial recovery. Post-COVID condition, also known also known as Long-COVID is defined as having a history of probable or confirmed SARS-CoV-2 infection, and persistent symptoms three months from the onset of COVID-19 [161]. It is characterised by cognitive impairment, post exertional malaise, pronounced fatigue, sleep disturbances, olfactory and gustatory disturbances and various other impairments [179]. Long-COVID can persist for months and even for years. The impact of Covid-19 on HRQoL after Acute or Long Covid patients is substantial, although impact on patients by gender, age, severity of illness and study country is disproportional [130].

**Fig. 1:**
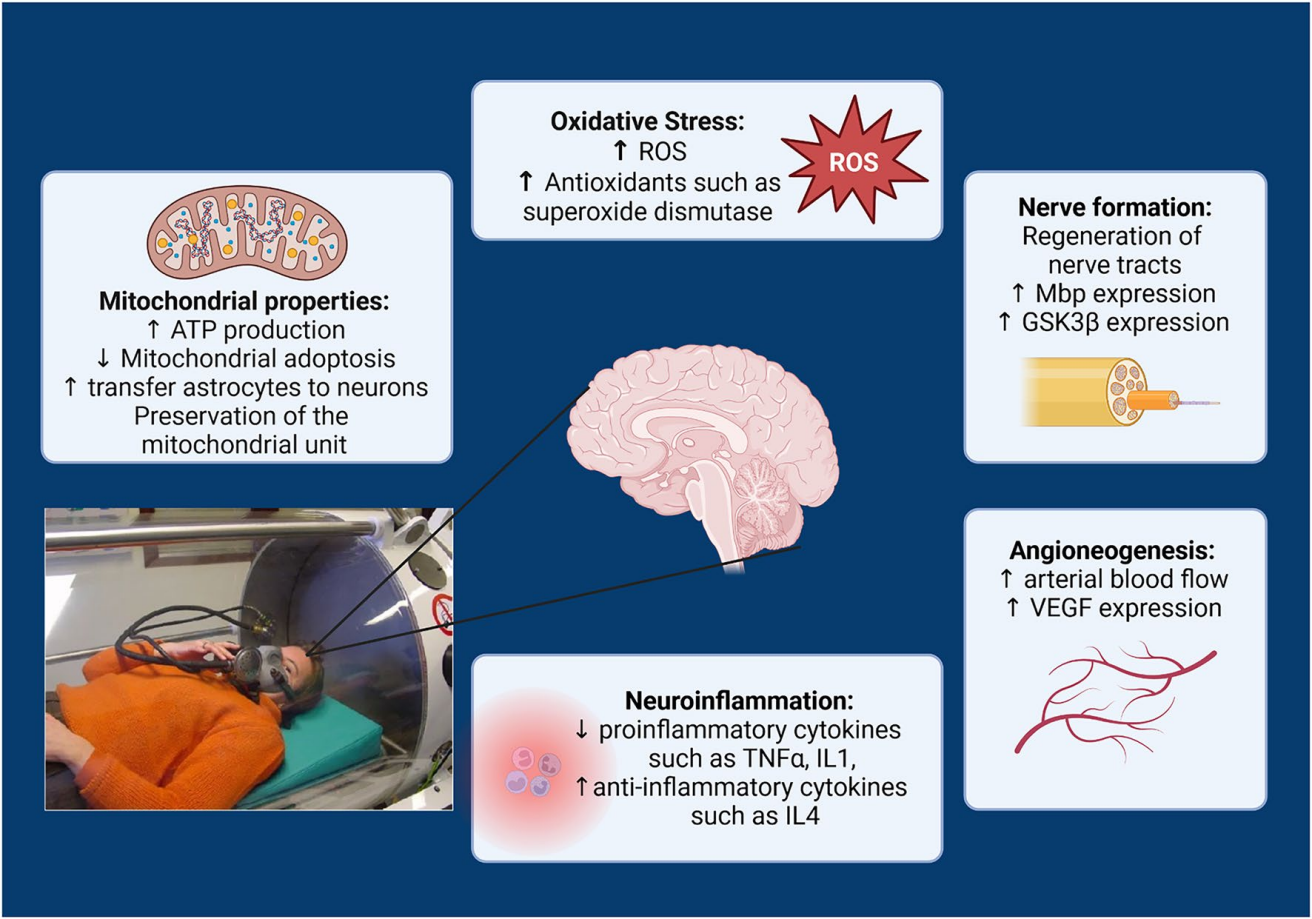
HBOT and it potential effects on different organs and tissues in Post-COVID syndrome

Patients with severe COVID-19 symptoms requiring hospitalisation are more likely to have more severe objective findings caused by organ injury than those with a mild course of the disease [77]. Although much has been tried clinically, an effective cure is not in sight yet despite promising therapy approaches [189]. Based on individual successful interventions with hyperbaric oxygen therapy (HBOT) in the acute phase of SARS-CoV-2 infections, we seek to elucidate the underlying pathophysiology regarding the potential targets of HBOT. Long-lasting post-COVID symptoms threaten individuals, populations and economies with 3 million in Germany and 144 million worldwide [121, 167, 181]. In a sample of 80 million people who underwent symptomatic SARS-CoV-2 infection between 2020 and 2023, 54% showed persistent fatigue with physical pain, cognitive problems or respiratory problems three months after initial infection [175, 181]. Field studies showed that among Italian COVID-19 patients, 53% reported fatigue and 22% reported chest pain after two months [140]; in a British cohort of 100 COVID-19 survivors, more than two-thirds of them had persistent fatigue after 4–8 weeks [187]. In addition, Greenhalgh et al. described a number of patients with weaker symptoms after viral infection due to orthostatic intolerance [56]. Similar to chronic fatigue syndrome, pro-inflammatory components through cytokines such as TFN-α and IL-7 are also thought to impair the regular function of the central nervous system in post-covid syndrome [35, 185], which can lead to a variety of the above-mentioned symptoms. Post-infection syndromes are basically not unknown and have been described for decades in other diseases such as influenza, polio or Epstein-Barr virus infections (post-influenza/post-polio syndrome) [60, 71, 136]. So far, however, there is no pathophysiological evidence for an independent clinical picture that could be proven by imaging or laboratory tests. Hyperbaric oxygen therapy is applied in several neurological diseases and syndromes with chronic fatigue or cognitive dysfunctions and it would be obvious to apply this therapy option to post and long COVID patients (Fig. 1) [30, 55, 160]. Based on the current concepts of Post Covid pathophysiology, this manuscript will attempt to bring the potential mechanisms of action of HBOT in other neurological diseases into an explanatory approach to POST COVID, incorporating the current clinical studies of HBOT.

## Method section

The search strategy to find adequate hyperbaric studies consisted the data bases of Cochrane Central, PubMed.gov, ClinicalTrials.gov and Web of Science. A search limit was set for articles published between 07/2020 and 31/07/2024. This period of time was set because COVID-19 pandemic started in December 2019. The post infection period with the emergence of Post COVID and Long COVID was visible from the second half of year 2020. The following search terms were used: “hyperbaric oxygen” AND “Long-COVID” OR “Post-COVID” OR SARS-COV-“infection.

## Current pathophysiological concepts of Long-COVID

Many different hypotheses on how long-COVID develops have been suggested [36, 132]. As far as HBOT has a potential therapeutic approach, we briefly outline the hypothesis and effect sites:

### Microvascular clotting and endothelial dysfunction [67]

COVID-19-induced endothelitis and dysfunction is also caused by an increase of endothelial adhesion molecules like selectins (e.g. ICAM-1), pro-inflammatory cytokines (e.g. IL-6-receptor and TNF-α), and pro-inflammatory chemokines (MCP-1), which may be released by activated and dysfunctional endothelial cells. These mediators could participate in leukocyte recruitment in the microvasculature. Pons et al. emphasized in a systematic review that in severe SARS-CoV-2 infections, endothelial cells play an important role of endothelial dysfunction in the vasculature, immunothrombosis and inflammation [129]. Hyperbaric oxygen is a clinical treatment that contributes to wound healing by increasing fibroblasts proliferation, collagen synthesis, and production of growth factors, inducing angiogenesis and inhibiting antimicrobial activity. It also has been shown that HBOT, through the activation of nitric oxide synthase promotes an increase in the nitric oxide levels that may improve endothelial progenitor cells (EPC) mobilization from bone marrow to the peripheral blood and stimulates the vessel healing process. However, cellular mechanisms involved in cell proliferation and activation of EPC after HBOT treatment remain unknown. A former publication from Cardenas et al. analyzed the effect of HBOT on the proliferation of pre-treated bone marrow-derived EPC with TNF-alpha. Also, they investigated the expression of ICAM and endothelial NO-synthetase (eNOS) by immunochemistry, the production of reactive species of oxygen and performed an in vitro wound healing. Although one hour of HBO treatment did not alter the rate of in vitro wound closure or cell proliferation, it increased eNOS expression and decreased ICAM expression and reactive oxygen species production in cells pre-treated with TNF-alpha. These results indicate that HBOT decreases the inflammatory response in endothelial cells mediated by TNF-alpha, and thus, promote vascular recovery after injury [16]. HBOT seems to improve vascular endothelial dysfunction in patients undergoing coronary stent implantation in a retrospectiv analysis. Patients showed siginificant differences after treatment compared to non-HBOT patients with significantly higher levels of FMD, NO and CGRP in the HBO2 group than those of the control group. Moreover, the high sensitive-CRP and ET-1 levels were significantly lower than those of the control group. Using HBO2 therapy as an adjunct treatment in patients undergoing coronary stent implantation may significantly improve vascular endothelial function [92].

### Dysfunctional signaling in the brainstem and/or the vagus nerve

Untari et al. could show that HBOT has been known to be common in illnesses and some clinical diseases [172]. Although it has not been indicated for peripheral neuropathy, a remarkable recovery was soon visible in the HBO-treated acute motor axonal neuropathy (AMAN) is a rare variant of Guillain-Barré syndrome and a paralytic disorder of abrupt onset characterized pathologically by motor nerve fiber degeneration of variable severity and by sparing of sensory fibers. There is little demyelination or lymphocytic inflammation [66]. The HBO mechanisms involved here are anti-inflammation and immunomodulation [172]. The mechanisms involved in HBOT-induced vasculoprotective effects are multifaceted. Cellular and molecular mechanisms of vascular aging such as blood brain barrier permeability, increased inflammation, mitochondrial dysfunction, oxidative stress, loss of Nrf2 activity, and NAD + depletion contribute to the pathogenesis of age-related cerebromicrovascular diseases [48, 55]. Growing evidence presented in this review suggests that HBOT targets these very same processes, ameliorating and reversing microvascular pathologies such as endothelial dysfunction, microvascular rarefaction, improved blood-brain-barrier features, mitochondrial function, cellular metabolism, inflammation, and oxidative stress, as well as ameliorating decreased NVC responses which contributes to the development of age-related neurodegeneration and VCID [9].

### Viral persistence of SARS-CoV-2

Patterson et al. reported data to support the hypothesis that an immune response to persistent viral antigens, specifically the S1-fragment of the spike protein, eliciting the PASC immune response [125] and marked by elevated inflammatory markers including IFN-γ, IL-6, IL-10, and IL-2, among others [124]. De Wolde et al. assessed neutrophilic reactive oxygen species (ROS) production after HBOT sessions in healthy volunteers. Specifically, they found systemic oxidative stress [plasma malondialdehyde (MDA) concentrations] as well as neutrophil phagocytic activity, plasma concentrations of tumor necrosis factor, interleukin-6, IL-8, and IL-10, and production of TNF, IL-6, and IL-10 by leukocytes ex vivo stimulated with the Toll-like receptor ligands lipopolysaccharide (TLR4) and Pam3Cys (TLR2). Decrease in neutrophilic ROS production and phagocytosis following the second HBOT session was found, which persisted after the third session, but no alterations in MDA concentrations [40]. Furthermore, plasma concentrations of the investigated cytokines were unaltered at all-time points, and ex vivo cytokine production was largely unaltered over time as well [37].

### Development of autoimmunity

Oxygenation and anti-inflammatory effects HBOT suggest to be a promising adjunct treatment for COVID-19. Repeated sessions of HBO with standard COVID-19 therapy were used to reduce the inflammation and increase oxygenation. Siewiera et al. evaluated the safety and efficacy of HBOT in avoiding the replacement ventilation and/or ECMO and its effect on the inflammatory process. Twenty-eight moderate-to-severe COVID-19 patients were randomized into control or HBOT group. HBOT patients participated in five hyperbaric sessions (60 min). Before and after each session blood gas levels and vital parameters were monitored. Blood samples were collected for extended biochemical tests, blood morphology and immunological assays. There were three deaths in the control, no deaths in the HBOT group. No adverse events leading to discontinuation of HBOT were observed and patients receiving HBOT required lower oxygen delivery. A decrease in CRP, ferritin and LDH and increase in CD3 in HBOT group was found compared to control group. This study confirmed the feasibility and safety of HBOT in patients with COVID-19 and indicated HBOT can lead to alleviation of inflammation and partial restoration of T-cell responses [157].

A distinction must be made between COVID-19 and other autoimmune diseases such as chronic inflammatory bowel diseases. In a meta-analysis, Singh et al. reported multiple mechanisms that have been proposed to explain the benefits of HBOT including hyperoxygenation, vasoconstriction, reduced leukocyte adherence, oxidative killing, promotion of angiogenesis and fibroblast proliferation, synergic effects on antibiotics and oxidative effects on bacteria. The results of their present meta-analysis showed a pooled clinical response rate of 83.3% when HBOT was used as an adjunct to standard therapy in active ulcerative colitis. Although pooled estimate suggests a high degree of benefit, the differences in available randomized studies with different population, co-intervention, treatment protocols and end points, preclude combining the data and to draw a definite conclusion on the benefit of adjunctive HBOT. Colectomy rates were 30% for ulcerative colitis after initial treatment with HBOT along with medical therapy. In Crohn’s disease, the pooled response rate was 81.9% when HBOT was used as an adjunctive therapy. In fistulizing Crohn’s disease, which is the severe form of this autoimmune disease, the pooled healing rates for complete response were 47% and for partial healing 34% in patients. In the setting of active ulcerative colitis, the summary estimates from the observational studies a good initial response with the use of HBOT. A cumulative response rate of 83% in ulcerative colitis flares was published. Although observational studies support the use of HBOT, the results remain to a certain degree conflicting, not least because of underpowering of study groups [158].

### Alteration of the microbiome in the intestine

Oxygen is a crucial element of human life as cell survival and operations depend on its sufficient occurrence in biological cells. It is probably not surprising that the SARS-CoV 2 virus can also impair the intestinal microbiome as a consequence after infection [98]. When human body becomes hypoxic, this critical condition affects the organs, tissues and cells and results in irreversible damage. Hypoxia may occur under various conditions, including both external environmental hypoxia and internal hypoxia. The gut microbiota play different roles under hypoxic conditions, and its products and metabolites interact with susceptible tissues. Han et al., described the changes of intestinal microbiota under different hypoxic conditions, both external and internal environment [98]. For external environment, altitude was the major cause inducing hypoxia. With increasing altitude, hypoxia will become more pronounced, and will affect also gut microbiota. On the other hand, internal hypoxia may occur in diseases like cancer, neonatal necrotizing enterocolitis and COVID-19 [21]. In addition to the diseases themselves, the occurring hypoxia may also change gut microbiota. The impact of localized hypoxia on the intestinal tract leads to lower oxygen content of the intestinal environment and creates a more favorable environment for intestinal anaerobic bacteria and will result in a higher risk of clostridioides difficile infection (CDI) [68].

### Multilocular organ and tissue damage [109]

As mentioned earlier, between 10 and 30% of patients are suffering from LC, but even a larger proportion of patients may suffer from organ damage without fulfilling the definition for LC. Multiorgan symptoms may affect the lungs, heart, gut, kidneys, liver, and brain [102] Ongoing inflammation may result in constitutional symptoms such as fatigue, brain fog, body aches, and organ specific dysfunction such as gastrointestinal dysregulation. Mainly symptoms of the cardiopulmonary system (e.g., dyspnoea, cough, atypical chest pain, and autonomic instability) and the neuropsychological (including neurological, cognitive, and psychiatric sequelae such as memory loss, executive dysfunction, depression, anxiety, severe fatigue, and sleep disruption) are observed in patients with Long Covid [166].

It is well known that traumatic limb injury is leading to direct tissue damage and additional local hypoxic conditions. The resulting edema causes acute traumatic peripheral ischemia. The trauma may vary from mild to irreversible and will involve major blood vessels and nerve injuries. For severe injuries, surgical amputations are sometimes necessarily performed. Vascular repair and reimplantation may be required for the limb to be viable. Examples of trauma include crush injury and thermal injury. Even without major vascular injuries, the tissue damage may lead to edema formation causing tissue hypoxia, which will lead to even more edema formation. This vicious cycle will lead to compartment syndrome within in poorly compliant muscle compartments, which then presents a surgical emergency to salvage the limb.

Critically perfused flaps also fall into the category of acute traumatic peripheral ischemia where hyperbaric oxygen has shown to improve ischemia. Surgical treatment and hyperbaric oxygen are not competing treatment modalities, but are best used to complement each other to provide the best outcome for the patient. Treating acute traumatic peripheral ischemia is one of the 13 approved hyperbaric oxygen indications by the Undersea and Hyperbaric Medical Society. It is also approved by the Centers for Medicare and Medicaid Services (CMS). The organization states that HBO therapy is valuable after acute traumatic peripheral ischemia or crush injuries and may supplementary applied in sutured severed where the loss of function, limb or life is existent [169].

### Immune dysregulation

One of the interesting and important findings in Post-COVID patients is the persistent dysregulation of the immune system. The major goal of each immune system is to react adequately with inflammation after trauma or infection. The balance between the initial inflammation with degradation of destroyed cells and tissues and the beginning of the reduction of pro-inflammation towards anti-inflammatory measures is an essential component of an adequate immune response. Symptoms in Long Covid patients resemble an auto-immune disease with chronic inflammation, dysregulated T cell activation and an increase in activated CD14+/CD16 + monocytes and plasmacytoid dendritic cells compared to healthy controls. In addition, significantly increased IFN-β and IFN-λ1 interferon levels are found in Long Covid. The combination of IFN-β, pentraxin 3, IFN-γ, IFN-λ2/3 and IL-6 was detected with an 8-month post-infection after SARS-Cov-2 [127].

Additionally, T cell dysfunction appears to promote the pathophysiology of long-COVID syndrome.

Autopsy studies of deceased COVID-19 patients showed that infiltrates in the lungs and other organs have elevated levels of CD8 + T cells. Elevated IL-6 levels have been observed in severe and moderate COVID-19 infection leading to inflammation and oxidative stress as a result of excessive reactive oxygen species (ROS) production and depleted antioxidant systems [57]. Since inflammation and oxidative stress are mutually reinforcing, the increase in IL-6 and ROS is the cause of permanent hyperinflammation in the body of long Covid patients [139]. In patients with necrotising fasciitis, a significant reduction in the proinflammatory cytokines IL-1, IL-6 and TNF-α could be measured with three days of HBO therapy, while the anti-inflammatory cytokine IL-10 showed an increase [69] .

### Damage caused by oxygen deficiency

**Fig. 2:**
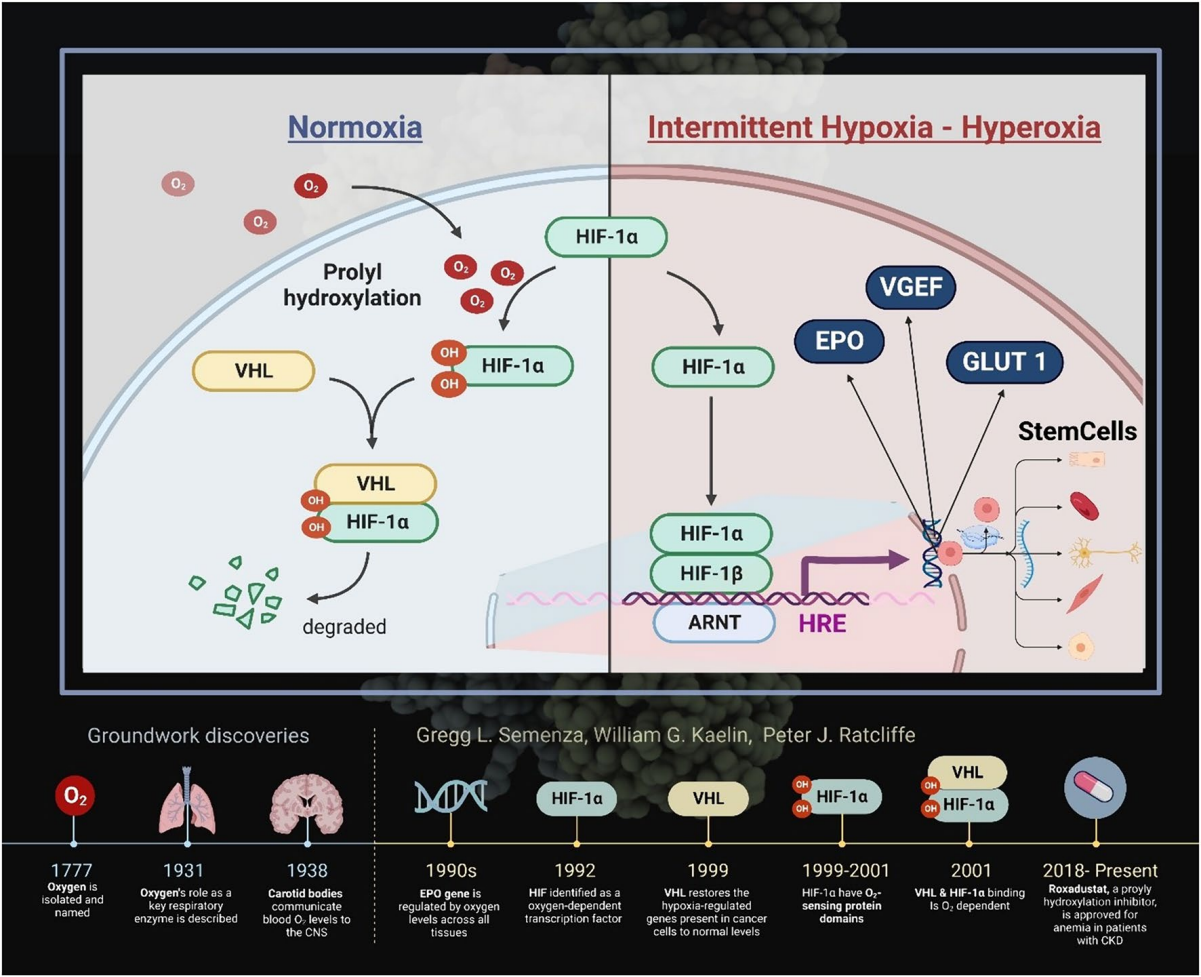
Hyperoxia-hypoxia paradox and HIF. The alternation of phases of hyperoxia with phases of normoxia induces the formation of numerous signalling molecules that contribute to the formation of angioneogenesis, wound healing and reparative processes

Maintaining a stable oxygen supply has been a crucial factor for aerobic organisms since the beginning of evolution. The partial pressure of oxygen in the body varies: it ranges from around 100–103 mmHg in the alveolar region of the lungs to around 1–2 mmHg at the cellular level in the mitochondria [114, 147]. In the process, 80% of the oxygen that reaches the cytosol is consumed by the mitochondria [23]. Interdisciplinary research by geneticists, nephrologists and oncologists led to the discovery that it is ultimately DNA segments (hypoxia response elements, HRE) that induce the cellular response in hypoxic conditions. In 2019, the fundamental findings that led to a better understanding of the molecular responses to cellular oxygen deficiency were awarded the Nobel Prize [148]. The focus is on the fact that oxygen is not only a central element in the energy supply of mammals, but also functions as a signaling molecule and regulates the concentration of the hypoxia-inducible factors in all cells of the body, not only in the kidney. The idea behind this is that when hypoxia occurs in the tissues, mechanisms are set in motion that ensure the provision of oxygen even in the case of severely reduced local oxygen availability, such as in alpine altitude regions, but also in the case of severe anaemia or excess oxygen demand such as exercise, inflammation and infections. HIF-1 is a dimeric transcription factor consisting of a continuously newly formed and oxygen-regulated α-subunit and a β-subunit constitutively expressed in the cell nucleus, the activity of which is not dependent on the oxygenation state [32]. The HIF-1α subunit is unstable under normoxic conditions and has a short half-life of 5 min [51]. With sufficient availability of oxygen molecules and/or ROS (oxygen radicals) that do not deviate greatly from the norm (physoxia), intracellular HIF-1α is rapidly degraded by prolyl hydroxylase enzymes that bind hydroxyl groups to 2 specific positions of HIF-1α. This prolyl hydroxylation allows the von Hippel-Lindau protein (VHL) to bind to HIF-1α and thus prepare it for degradation in the proteasome [149]. Under hypoxic conditions, the number of available oxygen molecules decreases. There is clear evidence of an accompanying increase in ROS, possibly originating from the respiratory chain. This situation now prevents the successful hydroxylation of HIF-1α, as a result of which this HIF subunit is stabilized and can combine with HIF-1β in the nucleus to form HIF-1, and then attach to the HRE binding site (Fig. 2).

In parallel, there is an analogous regulatory pathway for HIF-2α as well, both of which have a different share in the HRE binding site and activate different units of genes [28, 56, 80]. HIF-1α causes activation of glycolytic genes, induces apoptosis, and regulates cell pH through the activity changes of carbonic anhydrase. In contrast, HIF-2α regulates angiogenesis, erythropoietin release. Both HIF-1α and HIF-2α besides promote the transcription of vascular endothelial growth factor (VEGF), which induces the proliferation of new endothelial cells and vessels [151]. Through a series of proangiogenic genes, HIF stimulates angiogenesis in hypoxic regions by stimulating angiopoietin production, platelet growth and fibroblast growth [150]. There is a complex interaction between HIF-1 and HIF-2 depending on degree and duration of hypoxia, including non-coding RNA (microRNA) which also includes a third HIF (HIF-3), this interaction is called the “HIF-shift” [78].

The HIF-1 response seems to be a relevant defense mechanism in viral infections. HIF1α has been shown to limit Human cytomegalovirus (HCMV) replication through metabolic regulation by suppressing the expression of indoleamine 2, 3-dioxygenase 1 (IDO1), the rate-limiting enzyme in kynurenine synthesis [182]. The typical response in severe COVID-19 is a polarization of the innate immune response including micro glia towards the M1/ state, with excessive levels of proinflammatory cytokines, including IL-1β and IL-6, in moderate cases the dominant cells seems to be activated cytotoxic T-cells, all orchestrated by HIF-1 [94]. The HIF-1 response is triggered by hypoxia but is probably a defense mechanism, not only for cell survival but also for limiting viral spread since HIF-1α is shown to suppress ACE2-R and transmembrane protease serine 2 (TMPRSS2) [152]. The release of inflammatory cytokines and other metabolic changes causes increased vascular leakage and sooner or later brain or lung oedema and worsening hypoxia. The HIF-shift with prolonged hypoxia will upregulate HIF-2 and HIF-3 and attenuate the HIF-1 response [153]. This supports the assumption that inflammation induces hypoxia; one could even conclude that hypoxia and inflammation inevitably go hand in hand in the pathology of many diseases. HIF-1 is a potential target for attenuating immune dysregulation and endothelial dysfunction caused by COVID-19 [83, 87, 104, 144, 152].

## Virus properties of SARS-Cov-2

SARS-CoV-2 is a neurotropic virus with the ability to infect neuronal cell cultures such as brain organoids or rodent brains in experiments [24, 73, 116]. As a result, neuropsychiatric or neurological symptoms (e.g. cognitive and mental impairment, headaches, changes in smell and taste, fatigue and myalgia) may present themselves, as they do when infected with COVID-19 and other pathogenic human coronaviruses [56, 140, 187]. Even with mild COVID-19 infections, these neurological symptoms occur. We often see similar impairments in cognitive processes early on after COVID-19 infection [185]. While, on the one hand, SARS-CoV-2 RNA could be detected in the brain tissues of deceased COVID-19 patients in 30–40% of all cases [35, 101, 170], in other post-mortem examinations no corresponding histological changes or SARS-CoV-2 tissue were detectable in the brains of COVID-19 victims [22, 100, 173]. This would explain the so-called SARS-CoV-2 neurotropism but does not necessarily mean persistence of virus months or years after infection. The invasion of the nervous system of the SARS-CoV-2 virus is triggered via the angiotensin-converting enzyme 2 receptor (ACE2-R), which is expressed in the upper respiratory tract, but also in the brain stem and many other organs, including immune cells [133, 141, 155]. It should be noted that experimental studies show that SARS-CoV-2 can infect immune cells, with preference to activated T-cells in an ACE2-R-independent manner and cause apoptosis of T-cells associated with mitochondria-ROS-hypoxia pathways [155]. SARS-CoV-2 RNA have been found in various immune cell types, including neutrophils, macrophages, plasma B cells, T cells, and NK cells [134]. If immune cells are infected and used as Trojan horses in COVID-19 it may explain the complex and diverse symptomatology, the hypotheses dysregulated immune response, autoimmunity, reactivation of latent herpes virus and viral persistence depending on what subsets of immune cells that were infected. In a study analysing tissue from 21 different brain regions of deceased people, high ACE2 expression was found in the cerebral cortex, amygdala and brainstem [171, 180]. Pons and medulla oblongata of the brain stem showed the highest expression rate of ACE2-R. In addition to the ACE2-R, neuropilin-1 was also discovered as a co-receptor, which is mainly expressed in the olfactory nerve, where the concentration of ACE2-R is significantly lower [110]. Interestingly, however, neuropilin-1 is also expressed in the brainstem of juvenile animal brains, suggesting that the mature brainstem may also express neuropilin-1 and thus also provide a clinical aggravation of SARS-CoV-2 infection [133]. Pathogenic severe acute respiratory syndrome (SARS) and Middle East respiratory syndrome (MERS) have also been shown to often result in brainstem damage [91, 107, 165].

Neuroaffinity invasion of SARS-CoV-2 via the olfactory nerve into the brainstem can be demonstrated. SARS-CoV-2 RNA can be detected in 50% of brainstem samples and spike proteins in 40% of brainstem samples [105]. The brainstem belongs to the evolutionally ancient parts of the brain and includes the midbrain (mesencephalon), bridge (pons) and medulla oblongata. There are also indications in humans of a predilection of the brainstem, but not of a cerebrum infestation: SARS-CoV-2 RNA has not yet been detected there in deceased COVID-19 patients [2, 38, 44]. It can be concluded that SARS-CoV-2 could enter the brainstem via several pathways, including pathological immune or vascular activation. In particular, leukocyte infiltration, activation of microglia and astrocytes, and microthrombosis have been found in post-mortem sections of the brainstem [2, 15, 38, 99, 146]. The brainstem appears to be the locus for both acute and chronic diseases due to metabolic, vascular or other irritations [159]. Viral infection, inflammatory processes and vascular activation can also adversely affect brainstem function [93].

Subsequently, SARS-CoV-2 affinity to the brainstem is thought to be a likely cause of the impairment for respiratory and circulatory control, as vital cardiorespiratory neurons are located there [7, 31, 41, 50, 93, 183]. Furthermore, important gastrointestinal and neurological functions are also localised in the brainstem. This would also fit the diverse symptoms [19]. In various studies, the regenerative capacity of damaged peripheral nerves was delayed or even irreversible [25, 74, 194]. This suggests that brainstem damage following COVID-19 infection may also be irreversible. It is striking that the acute, viral infection and symptoms of Long-COVID are remarkably similar. About 30–50% of COVID-19 survivors suffer from respiratory symptoms such as chronic dyspnoea and cough for up to 24 months [5, 22, 103, 111, 154]. However, cardio-circulatory problems such as chest pain, palpitations and tachycardia are also common symptoms of long-term COVID and occur in about 20–40% of survivors [22, 143]. Another symptom appears to be postural orthostatic tachycardia syndrome (POTS) with several clinical subtypes [1, 112, 162]. All cardio-circulatory symptoms could be explained by the anatomically close relationship of neuronal pathways of the respiratory and cardiovascular neurons in the brainstem [14]. The brainstem also contains several important neuronal structures such as the raphe nuclei and the locus coeruleus, which are responsible for pain inhibition with their serotoninergic and noradrenergic neurons [76, 176]. In addition, these neurons supply anatomically more developed, cerebral regions with dopaminergic neurons [62, 99], which are associated with neurological disorders such as depression, anxiety, sleep and cognitive disorders, headaches, fatigue, myalgias and pain sensations [12, 123, 174, 188]. It may be assumed that a SARS-CoV-2 invasion of the brain stem leads to disturbances of these neurotransmitter structures, which would explain the symptoms described. Many different hypotheses on how long-COVID develops have been suggested [36].

## Hyperbaric oxygen therapy and its use

**Fig. 3:**
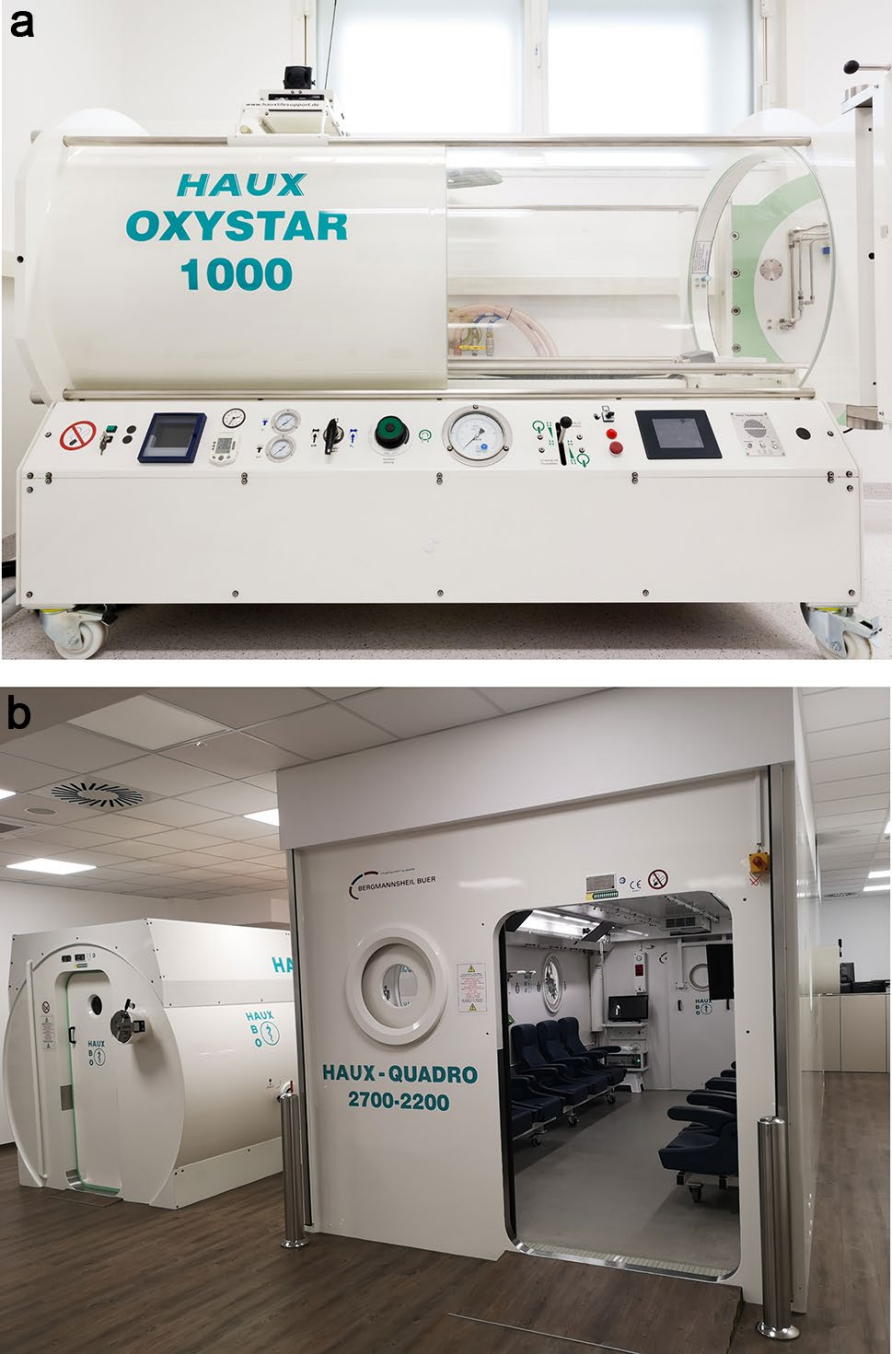
Monoplace and multiplace pressure chambers in use with patients. Monoplace pressure chambers can be operated with pure oxygen atmosphere as well as with ambient air. In the latter case, however, patients breathe oxygen through a mask in a closed system

**Fig. 4:**
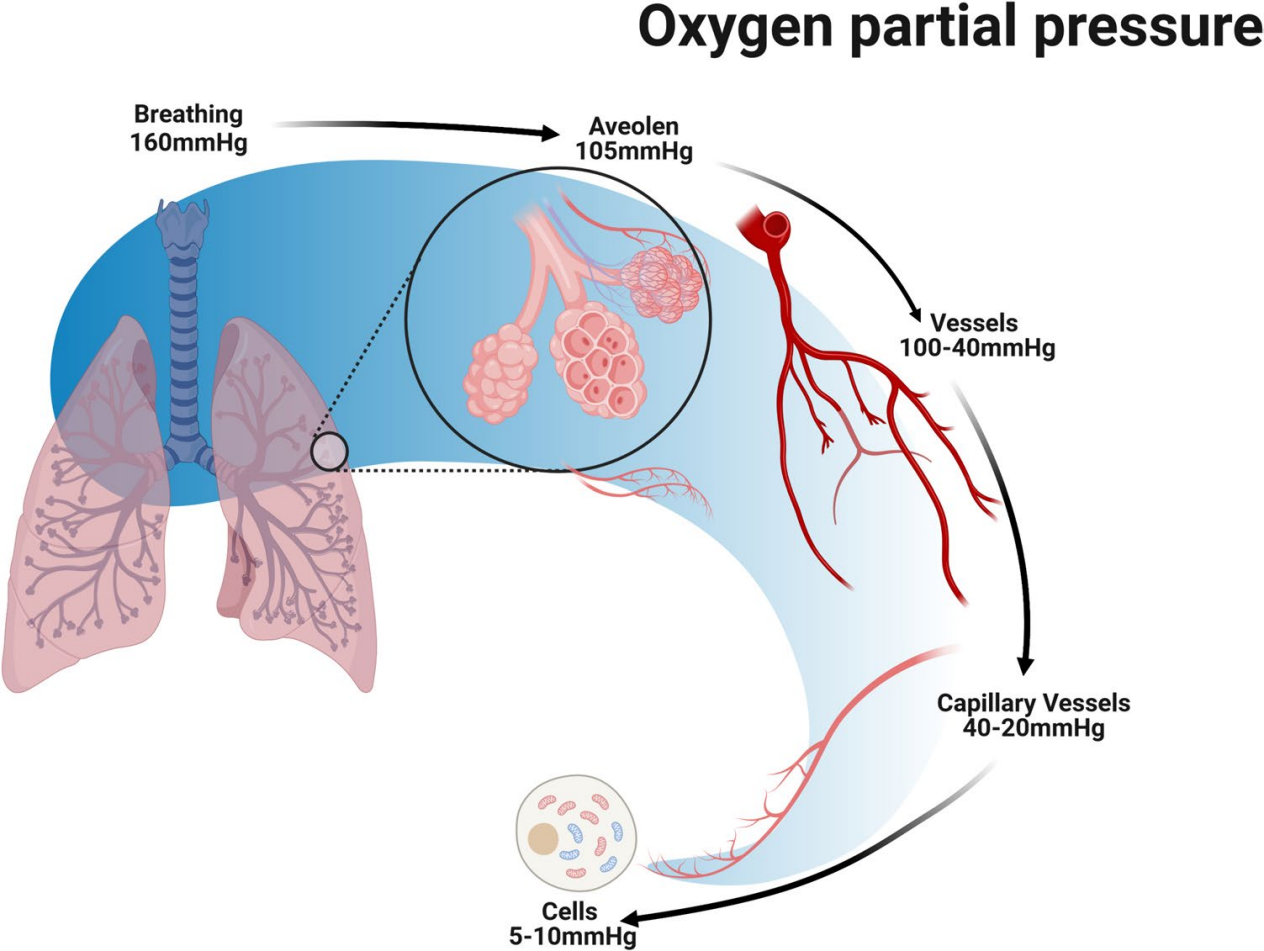
Oxygen concentrations through tissues. The signal transduction of HIF-1 for the detection of oxygen deficiency states in tissue. O_2_ partial pressure in the tissue is shown

The application of modern hyperbaric oxygen goes back to the Dutch heart surgeon Boerema [17]. Patients breathe pure oxygen under overpressure conditions totaling more than 2 bar in specially equipped pressure chambers (monoplace or multiplace pressure chambers) for different treatment times (Fig. 3). Under these conditions, the oxygen is physically dissolved in the blood in accordance with Henry’s law, independently of the haemoglobin, which is already almost 100% saturated under these conditions. Three to four times higher oxygen partial pressures (pO_2_ = 1800–2400 mmHg) are reached in the blood and tissue than under isobaric, pure oxygen respiration (pO_2_ = 500–600 mmHg). This also achieves four times higher diffusion distances than under normobaric oxygen breathing with the aim of achieving supranormal oxygenation in anaerobic tissues and mitochondria (Fig. 4).

In addition, the alternation of hyperbaric hyperoxia and returning normoxia after pressure chamber therapy leads to the induction of a number of effects at the cellular and molecular level. Current data suggest that under both hypoxia and hyperoxia conditions there are strong alterations in cellular ROS production, which in turn activates the HIF system under both conditions with corresponding activation of the HRE (hypoxia response element) (Fig. 2). It is not yet clear whether “relative hypoxia” after previous hyperoxia is the actual trigger event - the so-called “”Hyperoxia-Hypoxia Paradox (HHP)” [63]- or an increase in ROS production under both conditions, although significant hyperoxia does not occur under natural conditions [79].

In summary, the effects of HBOT include an improved oxygen supply [17], but also presumably HIF-dependent processes such as vascular endothelial growth factor (VEGF) [82], antiinflammatory effects [20], antioedematous effects [53], immunomodulating effects and the proliferation of various stem cell types [49, 72]. Furthermore, bactericidal and viricidal effects of hyperoxia are also suspected, as in the classic indication of clostridial myonecrosis [53].

**Fig. 5:**
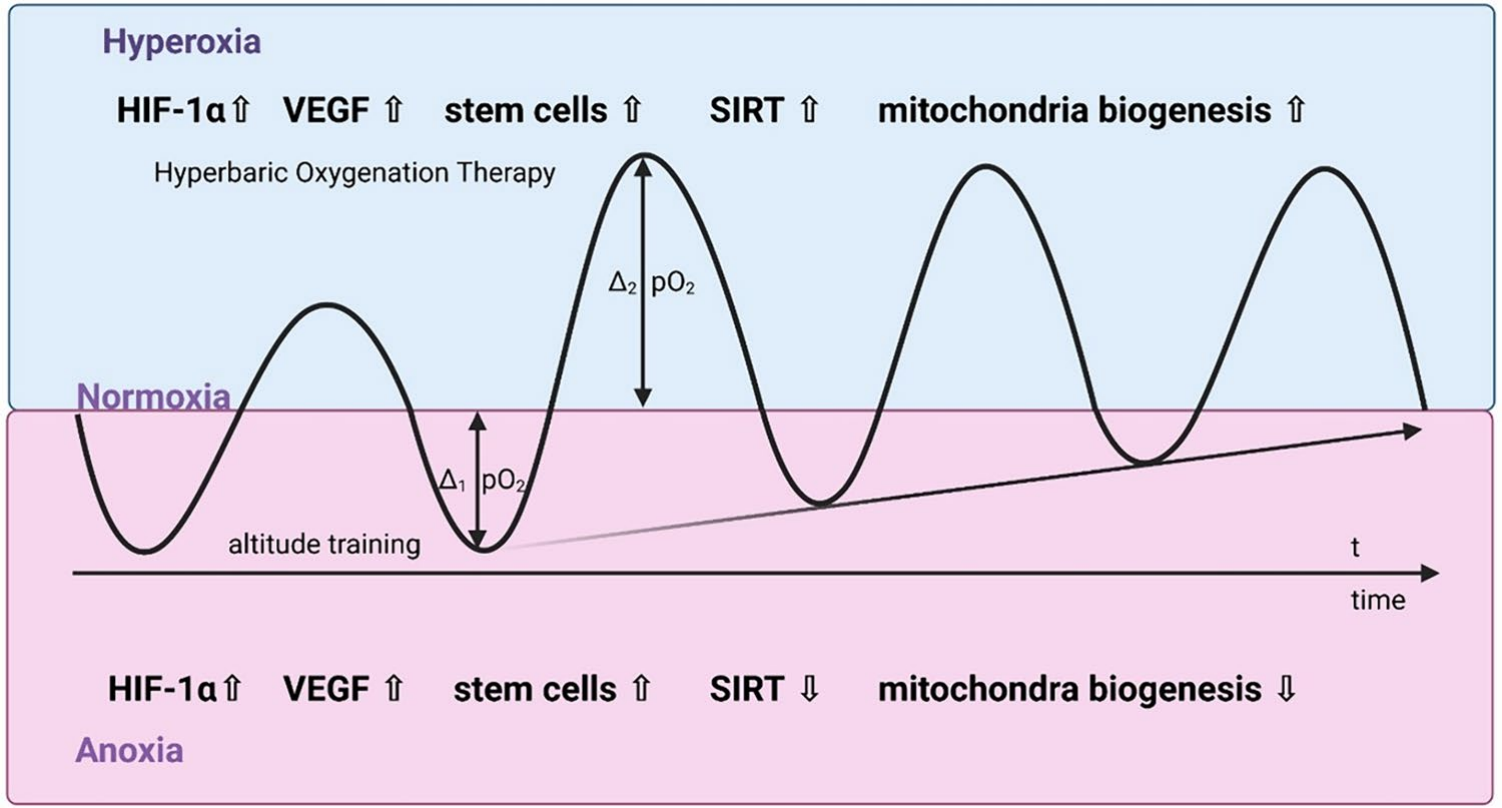
Effect of repetitive HBOT on different cytokines and cell messenger molecules. Intermittent hyperoxia and normoxia is imitating effects of altitude training, which leads from normoxia to hypoxia in tissues

At the cellular level, intermittent hyperoxia with oxygen partial pressures dropping at the end of normoxic values basically leads to a difference (Δ), as in the case of a drop in oxygen partial pressure due to a stay at alpine altitudes (Fig. 5). However, in contrast to the alpine environment patients are not at risk from the negative effects of “altitude sickness” such as cerebral or pulmonary oedema. Furthermore, intermittent hyperoxia will lead to stimulation of sirtuins (SIRT 1–6), a group of signaling molecules. They regulate mechanisms such as apoptosis, cell ageing and inflammation [6, 106, 131, 186, 191]. The complex, mutual influence between oxygen- and redox-responsive signal transducers occurs via the SIRT1-HIF interaction [29, 95].

Long/post covid is suspected to be associated with mitochondriopathy. The formation of intact mitochondria, the so called “power plants of the cell”, is crucial for maintaining the integrity of most cells and probably also plays an important role in preserving brain function and thus in neurodegenerative diseases [40]. Mitochondrial biogenesis is a complex process that requires coordinative tuning of cellular and mitochondrial DNA. Neosynthesis or repair of defective mitochondria is an extremely energy-consuming process, which is provided by high oxygen concentrations as in HBO-induced hyperoxia [4, 34, 70]. During this neogenesis, the inner and outer mitochondrial membranes, mitochondrially encoded proteins and the replication of mitochondrial DNA (mt-DNA) are coordinated. Several cell signaling pathways highly regulate mitochondrial biogenesis, of which the AMP-activated kinase (AMPK)-PGC-1a axis and sirtuin-1 (SIRT-1)-PGC-1a are particularly important. AMPK is activated by physiological stimuli such as exercise, starvation and transient hypoxia [41–44]. There is a considerable overlap between the AMPK and HIF signaling pathways, as both induce responses to increased energy demand with both opposing effects and interaction depending on the context [1, 54].

After infection by SARS-Cov-2 mitochondria will lead to a shift from the mitochondrial oxidative phosphorylation to the cytosolic glycolysis [39]. Normally the pentose phosphate pathway is in equilibrium with glycolysis, which means that any increase in glycolysis, allows SARS-Cov-2 to increase production of nucleotides for replication [45]. This will decrease mitochondrial ATP [108] and leads to a rise in ROS, which will result in oxidative damage in the host cell [113].

Furthermore, an energy-deprivation-induced decrease in nicotinamide adenine dinucleotide (NAD+), decreases SIRT1 activity. This leads to a lower concentration of pVHL protein, an essential inhibitor of HIF-1, and thus to an increased formation of HIF-1. A decreasing SIRT1 concentration results in reduced mitochondrial biogenesis and causes age-related diseases [186]. Conversely, overexpression of SIRT1 promotes mitochondrial biogenesis through deacetylation associated with activation of HIF-1 [3]. Overexpression of SIRT1 or SIRT6 is protective in many disease models in mice, including cancer, type 2 diabetes and cardiovascular disease [11, 47, 106, 131, 184] and leads to a delay in ageing and extension of lifespan [145].

Stem cells are cells that are able to generate a copy of themselves through asymmetric division and simultaneously form a specialized cell type through differentiation. Important stem cell lines are haematopoietic stem cells (HSC), immune cells, basal cells and mesenchymal stem cells (MSC). Neural stem cells (NSC) can form nerve cells and their glial cells such as oligodendrocytes and astrocytes throughout life [46] but also have inflammation-inhibiting effects that can be used for certain diseases [47]. Under normal conditions, stem cells are in a reversible state of quiescence, i.e. a temporary cell cycle arrest [48]. In the resting state, they show improved stress resistance and increased survivability. Oxygen plays an important role in regulating stem cell proliferation and differentiation [48–50]. Short-term hypoxia only can stimulate proliferation, migration and differentiation ability of stem cells and increase paracrine activity of mesenchymal stem cells [48, 50]. This induces an increased release of stimulating factors such as VEGF and exosomes, leading to increased vascularisation and anti-inflammation [50–52]. The effects of hypoxia on cells are predominantly regulated by increased expression of HIF-1 and the associated downward cascade of proteins [48, 50].

HBOT on the other hand is able to increase stem cell mobilization in various tissues [13, 46, 96] by affecting oxygen and pressuresensitive genes, which is promoting regenerative processes, such as stem cell proliferation and neurogenesis [126, 192]. This suggests that hyperbaric oxygen therapy may induce neuroplasticity and subsequently improve cognitive function [43, 52–55, 64].

## Studies and data to date

During the acute phase of the COVID-19 pandemic, the first applications of HBOT in patients with impending respiratory failure were made to break through the generalised inflammation by hyperoxic application of O_2_ and to achieve intermittent normoxic phases in the patient [84, 156]. Subsequently, first case reports reported on indications of a possible improvement of post-covidual symptoms [137]. A total of 10 consecutive patients received 10 HBOT sessions at 2.4 atmospheres over a period of 12 days. Validated assessments of fatigue and cognitive ability were conducted at day 1 and 10. HBOT resulted in statistically significant improvement in the Chalder fatigue scale, global cognition, executive function, attention, information processing and verbal function. A small observational study with *n* = 6 reported that all patients saw improvements in the measured symptoms to levels that were the same as pre-infection levels (five of six patients) or had significant improvement in symptoms (one patient). The results suggest that HBO2 helped to improve symptom scores, reduce the length of time of symptoms, and improved the quality of life [190].

**Fig. 6:**
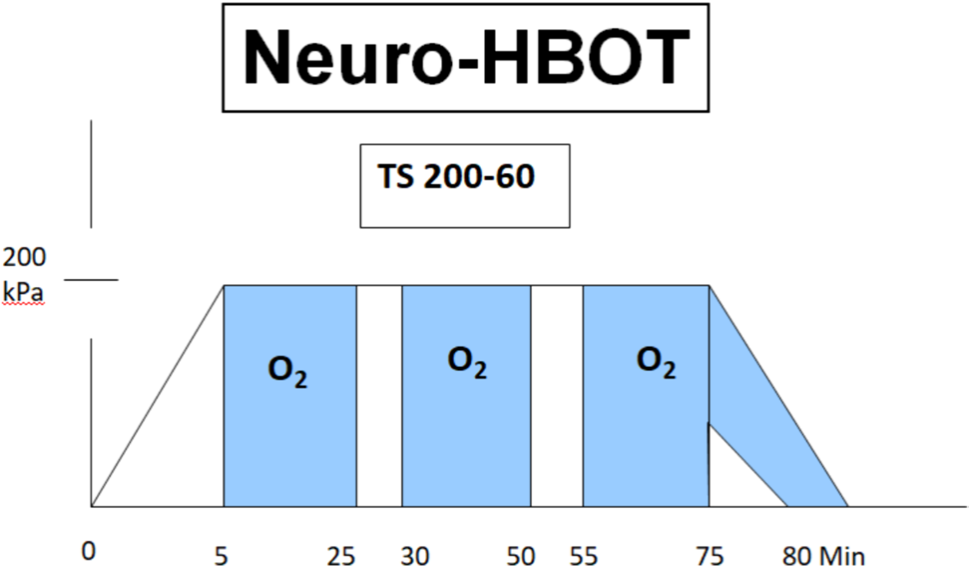
HBO neurology regimen (TS 200/90) as used in the Long-COVID study from Israel [199]. The total pressures (200 kPasc) are shown. The total pressure remains below the wound regimen (TS 240/90), which is most commonly used as in diabetic foot syndrome and wound healing disorders

Up to date, there is only one randomized, sham-controlled, prospective study with 73 patients from Israel that showed positive effects on some important symptoms of Post-Covid patients after the application of a total of 40 sessions at 2 ATA with air breaks (five sessions per week within a period of two months) [193]. The HBOT protocol involved the application of 100% oxygen by mask at 2 ATA for 90 min with five-minute air breaks every 20 min (Fig. 6). Hyperbaric oxygen was shown to induce structural and functional repair of damaged brain regions and improve cognitive, behavioral and emotional function in patients with long COVID. In addition, HBOT has been shown to improve white matter (neuronal fibre) attributable dysfunction and alter the functional connectivity organization of neuronal pathways significant for cognitive and emotional processes in patients after COVID-19 [26]. In the study group receiving HBOT, there were significant improvements in global cognitive function, attention and so-called executive function of the brain. In addition, there were also clinical improvements in the patients’ subjective energy levels, sleep and psychiatric symptoms. Interestingly, fMRI showed a correlation between the improvements and a significant increase in blood flow in several areas of the brain [27]. Microstructural changes in the supramarginal gyrus, the left supplementary motor area, the right insula, the left frontal precentral gyrus, the right middle frontal gyrus and the superior radial corona were detected. These results suggest that hyperbaric oxygen therapy can induce neuroplasticity and improve cognitive, psychiatric, fatigue, sleep and pain symptoms in patients suffering from post-COVID-19 disease. The positive effect is presumably due to increased brain perfusion and neuroplasticity in the affected regions with cognitive and emotional impairment. The same study population was evaluated by Leitman et al. in 2023 on possible suffer from cardiac dysfunction and are at increased risk for a broad range of cardiovascular disorders. Patients underwent echocardiography at baseline and 1–3 weeks after the last protocol session. Twenty-nine (48.3%) patients had a reduced global longitudinal strain (GLS) at baseline. Of them, 13 (43.3%) and 16 (53.3%) were allocated to the sham and HBOT groups, respectively. Compared to the sham group, GLS significantly increased following HBOT (−17.8 ± 1.1 to −20.2 ± 1.0, *p* = 0.0001), with a significant group-by-time interaction (*p* = 0.041). In conclusion, post-COVID-19 syndrome patients despite normal EF often have subclinical left ventricular dysfunction that is characterized by mildly reduced GLS. HBOT promotes left ventricular systolic function recovery in patients suffering from post COVID-19 condition [90]. Finally, Hadanny et al. [65], present a long-term evaluation of this study population with Participants were recruited more than one year (486 ± 73) after completion of the last HBOT session. Quality of life, assessed using the short form-36 (SF-36) questionnaire revealed, that the long-term results exhibited a similar magnitude of improvement as the short-term outcomes following HBOT across most domains. Regarding sleep quality, improvements were observed in global score and across five sleep domains with effect sizes of moderate magnitude during the short-term evaluation, and these improvements persisted in the long-term assessment [effect size (ES1) = 0.47–0.79]. In the realm of neuropsychiatric symptoms, as evaluated by the brief symptom inventory-18 (BSI-18), the short-term assessment following HBOT demonstrated a large effect size, and this effect persisted at the long-term evaluation. Both pain severity (ES1 = 0.69) and pain interference (ES1 = 0.83), had significant improvements during the shortterm assessment post HBOT, which persisted at long term. The results indicate HBOT can improve the quality of life, quality of sleep, psychiatric and pain symptoms of patients suffering from long COVID. The clinical improvements gained by HBOT are persistent even 1 year after the last HBOT session [65].

Another small observational study with *n* = 10 reports that HBO showed a decrease in fatigue in patients with long COVID, which could be explained by an improvement in energy metabolism and mitochondrial function by increasing tissue oxygenation. The cognitive improvement in the speed of information processing, an aspect valued by SDMT, would be supported by the increase in hyperoxygenation in the blood with an increase in cerebral blood flow, by the decrease in the presence of oxygen free radicals and various biomarkers of inflammation, as well as by brain neuroplasticity [52].

A publication from Austria with *n* = 59 and a three-month follow-up period draws the following conclusions: In total, 59 patients (33 females, 26 males; mean age: 43.9 years; range: 23–74 years; median: 45.0) were evaluable. After HBO, a statistically significant improvement of physical functioning (*p* < 0.001), physical role (*p* = 0.01), energy (*p* < 0.001), emotional well-being (*p* < 0.001), social functioning (*p* < 0.001), pain (*p* = 0.01) and reduced limitation of activities (*p* < 0.001) was confirmed. Physical functioning and both the physical and emotional role improved significantly and sustainably, suggesting HBO as a promising supportive therapeutic tool for the treatment of LCS [97].

A second randomized, placebo-controlled trial is currently being conducted in 80 patients in Sweden, the results of which are still pending [85, 86]. However, this is based on a different study concept, which only plans 10 HBOT with a total pressure of 2 bar. This sets the study apart from most centers that plan and perform between 15 and 30 HBOT.

In 2022, Kitala et al. [81] recruited 31 patients, 90% of whom were overweight or obese, and administered them with a cycle of 15 compression sessions. The results revealed a significant improvement in the feelings of anxiety, depression, and perceived pain. Using the Fullerton test, a positive effect on strength, fitness, and flexibility was reported. Moreover, significant changes in the anion gap, lactate levels, and postexercise saturation were reported. Furthermore, a statistically significant improvement in working memory and concentration was observed. Follow-up evaluation data demonstrated that improvements persisted even after the completion of HBOT. Regarding the parameters, the mean quality of life score was observed to be higher than the baseline score. Moreover, subjective experiences of improved physical performance, overall well-being, and energy levels, muscle or joint pain resolution, and improved sleep quality were reported. In 2023, conducted a one-group pretest–post-test study [115] with 15–50 HBOT sessions on five patients with fatigue, dyspnea, dry cough, and fever for 1–6 months following COVID infection. They evaluated the following numeric rating scales: reactive oxygen species (ROS) levels, total antioxidant capacity, cytokine concentrations from saliva samples, lipid peroxidation, DNA damage, NO metabolites, neopterin, creatinine, and uric acid concentrations from urine samples. The results revealed attenuated ROS production, lipid peroxidation, DNA damage, NO metabolites, and inflammation biomarker levels after HBOT [115]. A recent study conducted in Sweden was closed after recruitment of the planned patients. In this study, only 10 treatments were carried out, in contrast to the usual HBOT protocols of 20–40 treatments at 2.4 atmospheres. The results of this study regarding improvement of Long Covid symptoms are still pending; an interims safety report showed an unexpected high frequency of adverse events [86]. Actually there are still two HBOT studies running in Long Covid patients, one single center study in Germany (Berlin), and one multi-center study in the Netherlands.

## Discussion and perspectives

Long/Post Covid is a complex syndrome with a variety of symptoms and a pathology that is only partly understood. There are various hypothesis with different starting points for the patients. Beside direct virus effects on different human tissues the dysregulation of the immune system is crucial. The targeted application of oxygen by hyperbaric oxygen treatment may exert positive effects on the accompanying damaging inflammation. Moreover, HBOT treatment significantly reduces existing tissue hypoxia [10] and affects various transcriptional elements [178], which ultimately modulate cytokines such as IL-1β, IL-6 and TNF-α [8, 138, 164]. It can be deduced that HBOT treatment can at least attenuate cytokine-mediated inflammatory states. HBOT generates reactive oxygen species which, in addition to their potentially far-reaching controlling tasks in the HIF system and in inflammation control, also show additional virucidal effects [117, 122, 128]. COVID-19 infection appears to alter the 1-beta chain of hemoglobin, which can also lead to impaired oxygen uptake capacity [117]. This could be an additional argument in favor of HBOT treatment, as it can oxygenate the tissue by increasing the plasma oxygen delivery potential up to 20-fold [10]. The partial tension of oxygen (pO_2_) decreases along the oxygen cascade from the stimulated ambient air to the mitochondria, with oxygen serving as the final electron acceptor. A mild hyperoxic stimulus induces diversifications to counter the reduced oxygen availability [33]. An extreme acute respiratory distress syndrome, as can also occur with SARS-CoV-2 infection, can be understood as a response to damage to the mitochondria of the respiratory and circulatory structures due to direct viral damage and lead to pathological changes due to the induction of immune defense processes. The disturbed oxygen supply leads to changes that partly resemble changes in response to a mild hyperoxic stimulus. The aim of HBOT treatment is to improve blood flow and oxygen supply to the damaged tissue. HBOT treatment is also intended to increase arterial oxygen saturation and tissue oxygenation by improving cerebral microcirculation [18, 70]. In addition, HBOT has a crucial function in improving homeostasis at the matrix metalloproteases (MMP) through the blood-brain barrier [118, 119] and could additionally reduce intracranial stress and alleviate cerebral oedema [168]. However, secondary consequences of HBOT within the ischemic brain may induce a decrease in extracellular glutamate levels and lead to neural dysfunction up to excitotoxic death [58].

HBOT has been shown to provide oxidative protection against stroke-induced ROS and NOS [59]. This startling discovery refutes the fact that transporting excessive amounts of oxygen can seriously trigger oxidative stress and proves the effectiveness of HBOT. After stroke, HBOT has been shown to decrease the activity of pro-oxidant enzymes, including malondialdehyde, and increase it of CAT and SOD [75, 129]. In addition, other studies have reduced stroke-generated ROS within the striatum following HBOT treatment [163]. HBOT also has an influence on nitric oxide synthase and provides antioxidant protective properties [142]. However, due to the different experimental designs and the different treatment periods, it is not possible to obtain a complete picture of the function of HBOT in reducing oxidative damage. It is important to mention that HBOT treatment also represents an oxidative burden, but this is counterbalanced by endogenous antioxidant mechanisms within a short period of time [61].

In view of the large variety of symptoms in Post-Covid syndromes, it will be important to identify patients by means of suitable procedures that are as objectifiable as possible, such as standardized neuropsychological examinations, who could benefit from treatment with hyperbaric oxygen therapy [88]. From the perspective of the pathophysiological findings on Long/Post-Covid presented above and the physiological goals of hyperbaric therapy, clinical pictures in which oxygen deficiency states occur in the CNS and muscle tissue appear to be the most suitable. The known anti-inflammatory effects of hyperbaric oxygen could also show non-specific autoimmunological effects that could lead to an interruption of pathological, neuroimmunology processes. Stimulation of neural hypoxia, reduced NADH consumption in mitochondria and by HBOT could lead to regeneration of damaged CNS areas, as has been demonstrated in cerebral post-infarction syndromes [89]. In addition to the selection of suitable patients with a suitable underlying pathology, one focus will be the selection of suitable HBO protocols in terms of the number and duration of individual HBO sessions and the applied hyperbaric pressure. Based on clinical experience most HBOT centers would use regimens with at least 20–40 interventions in the last 50 years. It will be doubtful, whether a low number of applied HBOT sessions will be able to alleviate or even cure Long Covid symptoms. In this context, the results of the Swedish study, which is still pending, are awaited with great interest, but it is to be expected that the results will not be conclusive due to the low number of treatment interventions.

## Conclusion

HBOT seems to trigger regenerative effects through intermittent stimulation with hyperoxia, which may have a positive impact on the currently known pathomechanisms of a long/post-covid disease. Besides anti-inflammatory effects, HBOT shows improvements in oxygen supply in hypoxic areas and regeneration of nerve tissue through stimulation of neural stem cells. Up to this date HBOT with at least 40 sessions daily at 2 ATA is the only scientifically proven treatment in one prospective randomized controlled trial to be effective for long term benefical effects on cognitive improvement, regeneration of brain network and improvement of cardiac function. HBOT may have not only theoretical but also clinical impact on targets of current pathophysiology of Post COVID condition, which warrants further scientific studies in patients.

## Data Availability

To identify suitable research concerning the effects of hyperbaric oxygen on long-/post-covid syndrome, MTP searched the electronic database PubMed using three broad keyword combinations: (“hyperbaric oxygen”) AND (“long covid”) OR (“post covid”).

## Abbreviations

ACE2-R: Angiotensin converting enzyme 2-receptor
AMAN: Acute motor axonal neuropathy
AMPK: AMP-activated kinase
ATA: Atmosphere absolute
CAT: Catalase
COVID-19: Coronavirus disease 2019
EPO: Erythropoietin
HBOT: Hyperbaric oxygen treatment
HIF-1: Hypoxia inducible factor-1
LC: Long Covid
NOS: NO-synthetase
NRF2: Nuclear factor erythroid-2-related factor 2
NVC: Neurovascular coupling
fMRIF: Unctional magnetic resonance imaging
PASC: Post-acute sequelae of COVID-19
pVHL: Von Hippel-Lindau tumor suppressor
ROS: Reactive oxygen species
SARS-CoV-2: Severe acute respiratory syndrome coronavirus type 2
SIRT: Sirtuine
SOD: Superoxide dismutase
TNF: Tumor necrosis factor
TOLR: Toll like receptor
VCID: Vascular cognitive impairment and dementia
